# Highly Pathogenic Avian Influenza A(H5N8) Virus in Wild Migratory Birds, Qinghai Lake, China

**DOI:** 10.3201/eid2304.161866

**Published:** 2017-04

**Authors:** Mingxin Li, Haizhou Liu, Yuhai Bi, Jianqing Sun, Gary Wong, Di Liu, Laixing Li, Juxiang Liu, Quanjiao Chen, Hanzhong Wang, Yubang He, Weifeng Shi, George F. Gao, Jianjun Chen

**Affiliations:** CAS Key Laboratory of Special Pathogens and Biosafety, Chinese Academy of Sciences, Hubei, China (M. Li, H. Liu, J. Liu, Q. Chen, H. Wang, J. Chen);; Center for Influenza Research and Early-warning (CASCIRE), Chinese Academy of Sciences, Beijing (Y. Bi, G. Wong, D. Liu, Q. Chen, G.F. Gao, J. Chen);; Key Laboratory of Pathogenic Microbiology and Immunology, Chinese Academy of Sciences, Beijing, China (Y. Bi, G. Wong, D. Liu, G.F. Gao);; Shenzhen Third People’s Hospital, Shenzhen, China (Y. Bi, G.F. Gao);; Qinghai Lake National Nature Reserve, Qinghai, China (J. Sun, Y. He);; Northwest Institute of Plateau Biology of Chinese Academy of Sciences, Xining, China (L. Li);; Institute of Pathogen Biology, Taishan Medical College, Shandong, China (W. Shi)

**Keywords:** Highly pathogenic avian influenza virus, HPAI, H5N8, reassortment, wild aquatic birds, phylogenetic analysis, influenza in birds, bird diseases, viruses, influenza, wild migratory birds, Qinghai Lake, China

## Abstract

In May 2016, a highly pathogenic avian influenza A(H5N8) virus strain caused deaths among 3 species of wild migratory birds in Qinghai Lake, China. Genetic analysis showed that the novel reassortant virus belongs to group B H5N8 viruses and that the reassortment events likely occurred in early 2016.

Since 2003, the A/Goose/Guangdong/1/96 lineage (Gs/Gd-lineage) of highly pathogenic avian influenza (HPAI) A(H5N1) viruses has been evolving into diverse clades and subclades (*1*). A novel subclade of HPAI A(H5N8), 2.3.4.4, which evolved from a clade 2.3.4 H5N1 variant, was initially isolated from domestic ducks in eastern China in 2010 (*2*) and caused outbreaks in domestic ducks and migratory birds in South Korea in early 2014 (*3**,**4*). In late 2014, several countries in Europe and East Asia experienced an invasion of HPAI H5N8 virus (*5*). This HPAI H5Nx (H5N8, H5N2, and H5N1) lineage subsequently emerged in North America, causing fatalities among wild birds and outbreaks in domestic poultry (*5*). 

Available evidence strongly suggests that the HPAI H5N8 subclade 2.3.4.4 viruses were introduced and spread across the globe by migratory birds (*6**–**8*). Currently, 2 distinct H5N8 virus groups have been identified: group A (Buan2-like) and group B (Gochang1-like) (*3*). Group A H5N8 viruses predominate and have further evolved into 3 distinct subgroups: icA1, icA2, and icA3 (*6*). We report the emergence of a group B H5N8 virus in Qinghai Lake, China, a key breeding and stopover site for waterfowl along the Central Asian Flyway.

## The Study

**Figure 1:**
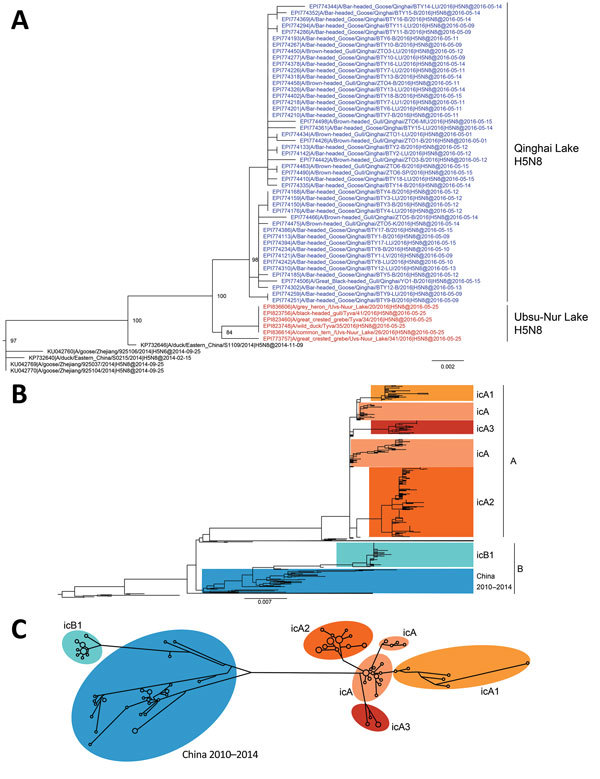
Phylogenetic analyses of 594 hemagglutinin (HA) sequences (1,704 nt) from clade 2.3.4.4 H5 influenza viruses. A) HA-coding sequence subtree from maximum-likelihood phylogenetic analysis of the clade 2.3.4.4 H5 viruses. Colored nodes: blue, Qinghai Lake H5N8 strains (this study); red, Ubsu-Nur Lake H5N8 strains. B) Maximum-likelihood phylogenetic tree of the clade 2.3.4.4 HA-coding sequences, rooted with A/Goose/Guangdong/1/96(H5N1). Scale bars indicate nucleotide substitutions per site. C) Median-joining phylogenetic network of the HA-coding gene sequences, including the most parsimonious trees linking the sequences. To simplify the network, nodes with only one sequence are not shown. Network branch lengths are proportional to the numbers of mutations. icA, intercontinental group A; icA1, intercontinental subgroup A1; icA2, intercontinental subgroup A2; icA3, intercontinental subgroup A3; icB1, intracontinental subgroup B1.

On May 1, 2016, the carcass of a brown-headed gull (*Larus brunnicephalus*) was found on Egg Islet, a major breeding site of bar-headed geese, in Qinghai Lake. Carcasses of wild birds were recovered for 15 consecutive days, starting on May 8: 124 bar-headed geese (*Anserindicus*), 17 brown-headed gulls (*Larus brunnicephalus*), and 14 great black-headed gulls (*L. ichthyaetus*). As of June 4, a total of 158 birds, most of which were bar-headed geese, were found dead in Qinghai Lake, predominantly on Egg Islet (Figure 1; Technical Appendix)

In the first 9 days of the outbreak, multiple organs (brain, intestine, liver, lung, pancreas and kidney) were collected aseptically from 18 bar-headed geese, 6 brown-headed gulls, and 1 great black-headed gull. We inoculated 10-day-old chicken embryos with the homogenates of these organs for virus isolation. Almost all organs analyzed were positive for influenza virus, and we detected only H5- and N8- subtype-specific strains.

We sequenced full-length genomes and found the polybasic amino acid sequence, REKRRKR*GL in the hemagglutinin (HA) cleavage site, confirming the virus can be classified as highly pathogenic. Sequences of 48 Qinghai Lake H5N8 influenza isolates (QH-H5N8) were deposited into the GISAID database (http://www.gisaid.org) under accession nos. EPI774110–EPI774510.

Sequence comparisons showed high nucleotide identity among all 8 gene segments of the QH-H5N8 isolates (>99.2 %; data not shown), indicating that the isolated strains are descendants of a common ancestral virus. A BLAST search (https://blast.ncbi.nlm.nih.gov/) suggested that QH-H5N8 is a reassortant virus (Technical Appendix Table 1) and that the HA, neuraminidase (NA), and nonstructural protein (NS) genes of QH-H5N8 share high nucleotide identity (>99.1%) with those of the H5N8 virus that circulated among poultry in eastern China in 2014 (A/duck/Eastern China/S1109/2014[H5N8]). The remaining internal genes share high nucleotide identity with those of the low pathogenicity avian influenza (LPAI) viral pool in waterfowl from Mongolia and other regions (Technical Appendix Table 1).

Phylogenetic analysis confirmed that the 8 segments had different origins. In the HA, NA, and NS phylogenetic trees, the QH-H5N8 virus clustered with H5N8 viruses isolated in late May 2016 from wild waterfowl at Ubsu-Nur Lake (UN-H5N8), forming a monophyletic cluster (Figure 1, panel A; Technical Appendix Figure 2, panels D, F, H). Unlike the H5N8 strains previously described in South Korea in 2014–2015 (Buan2-like, group A), this cluster fell within group B (Gochang1-like) H5N8 viruses, forming a novel subgroup, intracontinental group B (icB1) (Figure 1, panel B; Technical Appendix Figure 2, panel D). Neighbor-joining phylogenetic network analysis of the HA segment of the clade 2.3.4.4 H5 viruses also supported the finding that the QH-H5N8 and UN-H5N8 strains form a monophyletic cluster and appear to have evolved independently from group A H5N8 viruses (Figure 1, panel C).

Phylogenetic trees constructed by using sequences from the internal genes (all but polymerase basic 1 [PB1]) show that QH-H5N8 and UN-H5N8 viruses are closely related to various LPAI viruses circulating in aquatic birds in Mongolia in 2015. The PB1 gene, however, originated from various LPAI viruses dispersed across a relatively large geographic region (East and South Asia) over a long period (2010–2015) (Technical Appendix Figure 2).

**Figure 2:**
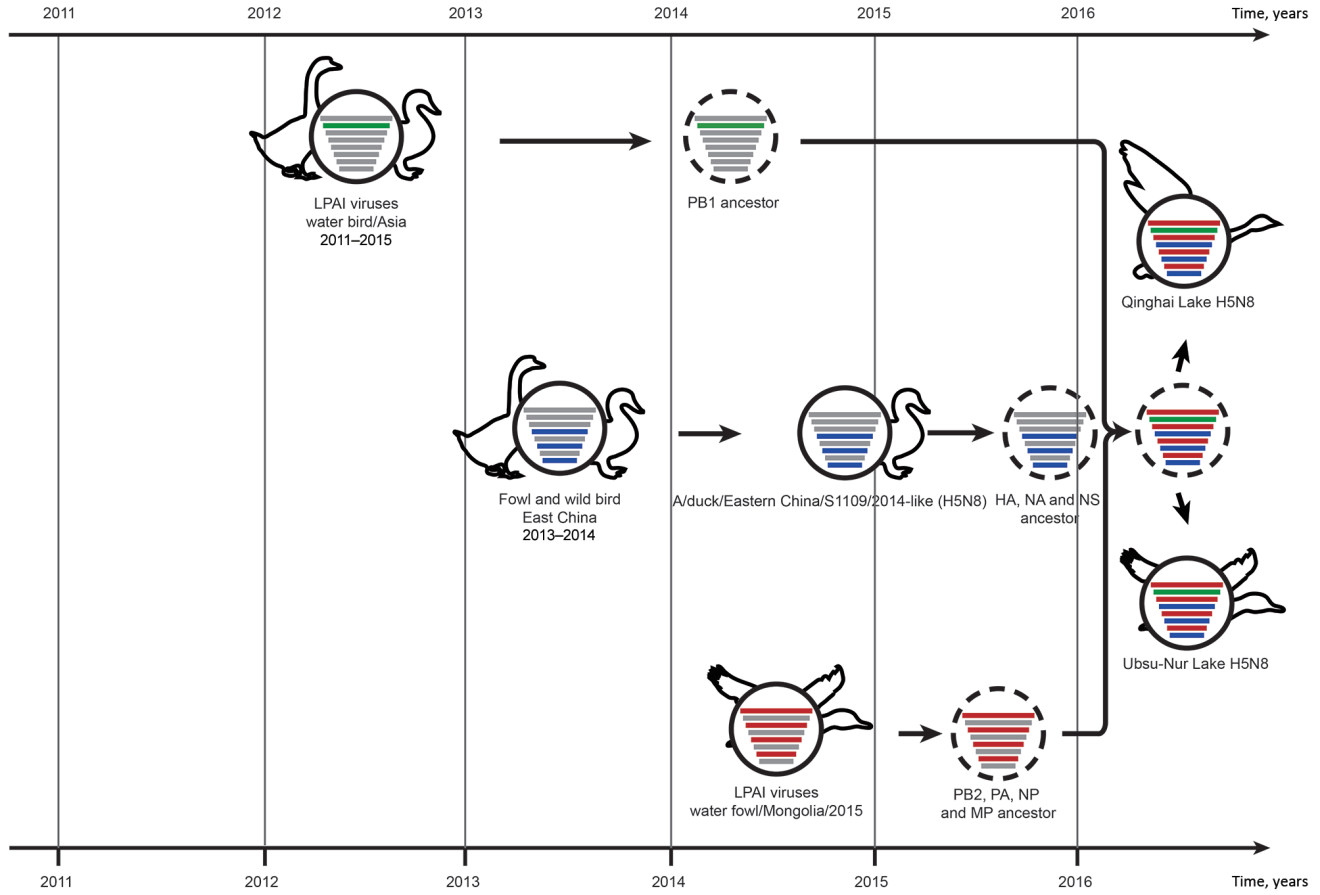
Hypothetical evolutionary pathway of influenza (H5N8) viruses from Qinghai Lake, China. Gene segments are colored according to their origins. Dashed virions indicate unidentified viruses. HA, hemagglutinin; LPAI, low pathogenicity avian influenza; MP, matrix protein; NA, neuraminidase; NP, nucleoprotein; NS, nonstructural protein; PA, acidic polymerase; PB, polymerase basic.

We used molecular dating to estimate the timing of the reassortment events that led to the emergence of QH-H5N8 (Technical Appendix Figure 3). The HA, NA, and NS genes were transferred from domestic waterfowl in eastern China to wild migratory birds in approximately October 2015, January 2016, and December 2015, respectively (Figure 2; Technical Appendix Table 2). Other internal gene segments (except PB1) originated from Mongolian waterfowl during July 2014–January 2016 (Figure 2; Technical Appendix Table 2, Figure 3). The PB1 segment differs from the other segments, and was transferred from a LPAI virus circulating among waterfowl in Asia in February 2014 (Figure 2; Technical Appendix Table 2, Figure 3). Thus, the generation of QH-H5N8 in wild migratory birds appears to have been a complex process and was likely completed in early 2016 (Figure 2).

## Conclusions

We show that a group B H5N8 virus emerged in Qinghai Lake, China, causing deaths in wild migratory birds. Phylogenetic analysis indicates that the QH-H5N8 virus is the descendant of an unidentified triple-reassortant strain (Figure 2). The reassortment event may have occurred in waterfowl, and can be traced back to early 2016. However, we cannot infer the geographic region where the reassortant virus was generated, because the gene constellation of the virus originated from different locations.

The absence of domestic poultry in the vicinity of Qinghai Lake strongly suggests that the virus was introduced to the area by wild birds. The deaths in Qinghai Lake occurred during May−June 2016, which corresponds with the breeding season for the affected species. In late May 2016, similar H5N8 strains were detected in wild migratory birds at Ubsu-Nur Lake, 1,600 km north of Qinghai Lake (*9**,**10*). This finding suggests that the early summer movement of wild migratory birds from unknown southern sites to northern breeding grounds resulted in the introduction of H5N8 to Qinghai Lake and to Ubsu-Nur Lake, infecting a diverse population of breeding waterbirds.

Currently, we know of 3 HPAI H5N1 virus clades that have been introduced to wild migratory birds in Qinghai Lake, which is located near multiple migratory flyways: clade 2.2 in 2005 (*11**,**12*), clade 2.3.2 in 2009 (*13**,**14*), and clade 2.3.2.1c in 2015 (*15*). On all 3 occasions, similar viruses were subsequently detected in other regions. Therefore, when wild birds left the breeding location for their wintering sites in the autumn of 2016, H5N8 virus could potentially have spread to other regions along the flyway. HPAI H5N8 viruses have already caused fatalities among wild birds or poultry in South Asia, Europe, the Middle East, and Africa (http://www.oie.int/) since late October 2016. Available genetic information shows that H5N8 strains isolated in other countries are highly similar to the QH-H5N8-like virus, suggesting that the QH-H5N8-like viruses may have already disseminated to other areas along the migratory flyways.

Technical Appendix Detailed methods, sequence homologies of the QH-H5N8 genome, Most Recent Common Ancestor results, phylogenetic analysis of gene segment sequences.
