# Supplementary material for: Highly Pathogenic Avian Influenza A(H5N8) Virus in Wild Migratory Birds, Qinghai Lake, China
Source: Emerg Infect Dis. 2017 Apr;23(4):637–41. doi: 10.3201/eid2304.161866 (PMC5367427; doi:10.3201/eid2304.161866)
Supplement: Technical Appendix — Detailed methods, sequence homologies of the QH-H5N8 genome, Most Recent Common Ancestor results, phylogenetic analysis of gene segment sequences. [file 16-1866-Techapp-s1.pdf]

Article DOI: http://dx.doi.org/10.3201/eid2304.161866

Highly Pathogenic Avian Influenza A(H5N8) Virus in Wild Migratory Birds, Qinghai Lake, China

Technical Appendix

Detailed Methods

Phylogenetic Analysis

We downloaded all influenza A virus genomes available in GenBank and the Global Initiative on Sharing All Influenza Data (GISAID) and combined them into a single database. We queried each nucleotide sequence of strain A/Bar-headed Goose/Qinghai/BTY1-B/2016 (H5N8) against the combined database by using the blastn program in National Center for Biotechnology Information (Bethesda, MD, USA) NCBI blast 2.2.29+ with default parameters and collected sequences of the top 500 hits. Sequences without a clear subtype and collection date were removed from each segment dataset. For HA and NA segments, additional sequences from H5N8 strains not included in the blastn result were appended to cover all available H5N8 strains. The final sequence numbers of each dataset were: basic polymerase 2 (PB2), 468; basic polymerase 1 (PB1), 466; acidic polymerase (PA), 458; hemagglutinin (HA), 594; nucleoprotein (NP), 424; neuraminidase (NA), 485; matrix protein (MP), 460; nonstructural protein (NS), 425. We used Clustal Omega 1.2.1 to generate coding sequence alignments of the 8 segments. The alignment lengths for each dataset were: PB2 2,277 nt , PB1 2,271 nt, PA 2,133 nt, HA 1,704 nt, NP 1,494 nt, NA 1,410 nt, MP 964 nt, and NS 835 nt. For each dataset, we used the GTR+G nucleotide substitution model in RAxML v.8.2.6 with 1,000 bootstrap replicates for phylogenetic analysis.

Molecular Dating

The time of the most recent common ancestor for each segment was estimated by Bayesian Evolutionary Analysis using Sampling Trees (BEAST v 2.3.2) on a smaller dataset

composed of Qinghai Lake strains and related virus strains, with the Hasegawa-Kishino-Yano plus invariant nucleotide substitution model and a relaxed clock. All chains were run in 50,000,000 generations with 10% burn-in, and all effective sample size values in the results were greater than 200. The maximum clade credibility phylogenetic trees were generated by TreeAnnotator in the BEAST package and visualized/annotated with FigTree 1.4.2 (http://tree.bio.ed.ac.uk/software/figtree/).

The neighbor-joining phylogenetic network of 594 H5 HA CDS sequences was generated by the software Fluxus network 5.0 (http://www.fluxus-engineering.com/sharenet.htm). Due to the complexity of the network, the options frequency was set to >1, and Star Contraction and MP were applied to simplify the resulting network.

Technical Appendix Table 1. Sequence homologies of the whole genome of the QH-H5N8 virus, with nucleotide sequences available in GenBank

Gene*	Viruses with the highest nucleotide identity (%) to QH-H5N8	GenBank accession no.	Homology, %
PB2	A/duck/Mongolia/30/2015(H3N8)	LC121233.1	98.6
	A/duck/Mongolia/655/2015(H2N3)	LC121425.1	98.5
PB1	A/duck/Mongolia/179/2015(H3N8)	LC121306.1	98.0
	A/mallard/Sweden/101900/2009(H4N3)	CY165926.1	97.8
PA	A/muscovy duck/Vietnam/LBM529/2013(H3N8)	AB916666.1	99.0
	A/duck/Mongolia/996/2015(H3N8)	LC121467.1	98.7
HA	A/duck/Eastern China/S1109/2014(H5N8)	KP732646.1	99.1
	A/goose/Zhejiang/925037/2014(H5N8)	KU042769.1	98.4
NP	A/duck/Mongolia/129/2015(H3N3)	LC132921.1	98.8
	A/duck/Mongolia/167/2015(H3N8)	LC121293.1	98.7
NA	A/duck/Eastern China/S1109/2014(H5N8)	KP732686.1	99.4
	A/duck/Eastern China/S0215/2014(H5N8)	KP732680.1	98.9
MP	A/duck/Mongolia/179/2015(H3N8)	LC121311.1	98.5
	A/duck/Mongolia/709/2015(H10N7)	LC121439.1	98.5
NS	A/duck/Eastern China/S1109/2014(H5N8)	KP732726.1	99.3
	A/goose/Yangzhou/0420/2014(H5N8)	KT221086.2	98.8

*PB2, basic polymerase 2; PB1, basic polymerase 1; PA, acidic polymerase; HA, hemagglutinin; NP, nucleoprotein; NA, neuraminidase; MP, matrix protein; NS, nonstructural protein.

Technical Appendix Table 2. Most recent common ancestor (MRCA) of each segment of the QH-H5N8 virus

Segment*	MRCA†	95% highest posterior density (HPD) range
PB2	2015, Oct	Aug–Dec 2015
PB1	2014, Feb	Jan 2013–Jan 2015
PA	2015, Oct	Aug 2015–Jan 2016
HA	2015, Oct	Jul–Dec 2015
NP	2015, Jan	Jul 2014–Jul 2015
NA	2016, Jan	Oct 2015–Mar 2016
MP	2015, Sep	Jun–Dec 2015
NS	2015, Dec	Sep 2015–Mar 2016

*PB2, basic polymerase 2; PB1, basic polymerase 1; PA, acidic polymerase; HA, hemagglutinin; NP, nucleoprotein; NA, neuraminidase; MP, matrix protein; NS, nonstructural protein.
†The time to the Most Recent Common Ancestor (MRCA), is estimated by using the molecular clock theory in Bayesian Evolutionary Analysis using Sampling Trees. This parameter represents the potential existing timing of a specific internal node.

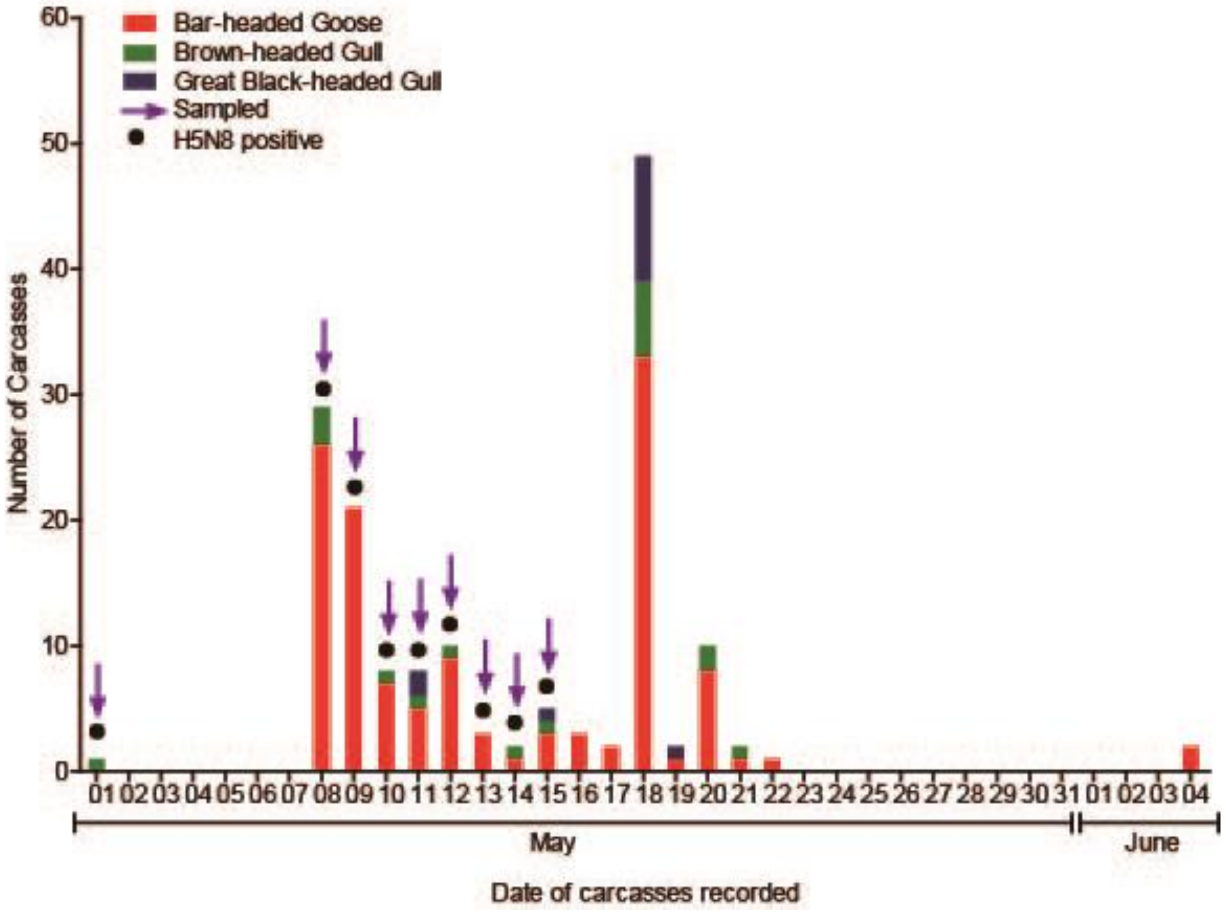

Technical Appendix Figure 1. Numbers and species of bird carcasses found in Qinghai Lake from May 1, 2016 to June 4, 2016. Purple arrows indicate sampling dates and black circles indicate H5N8 virus positivity.

Technical Appendix Figure 2A

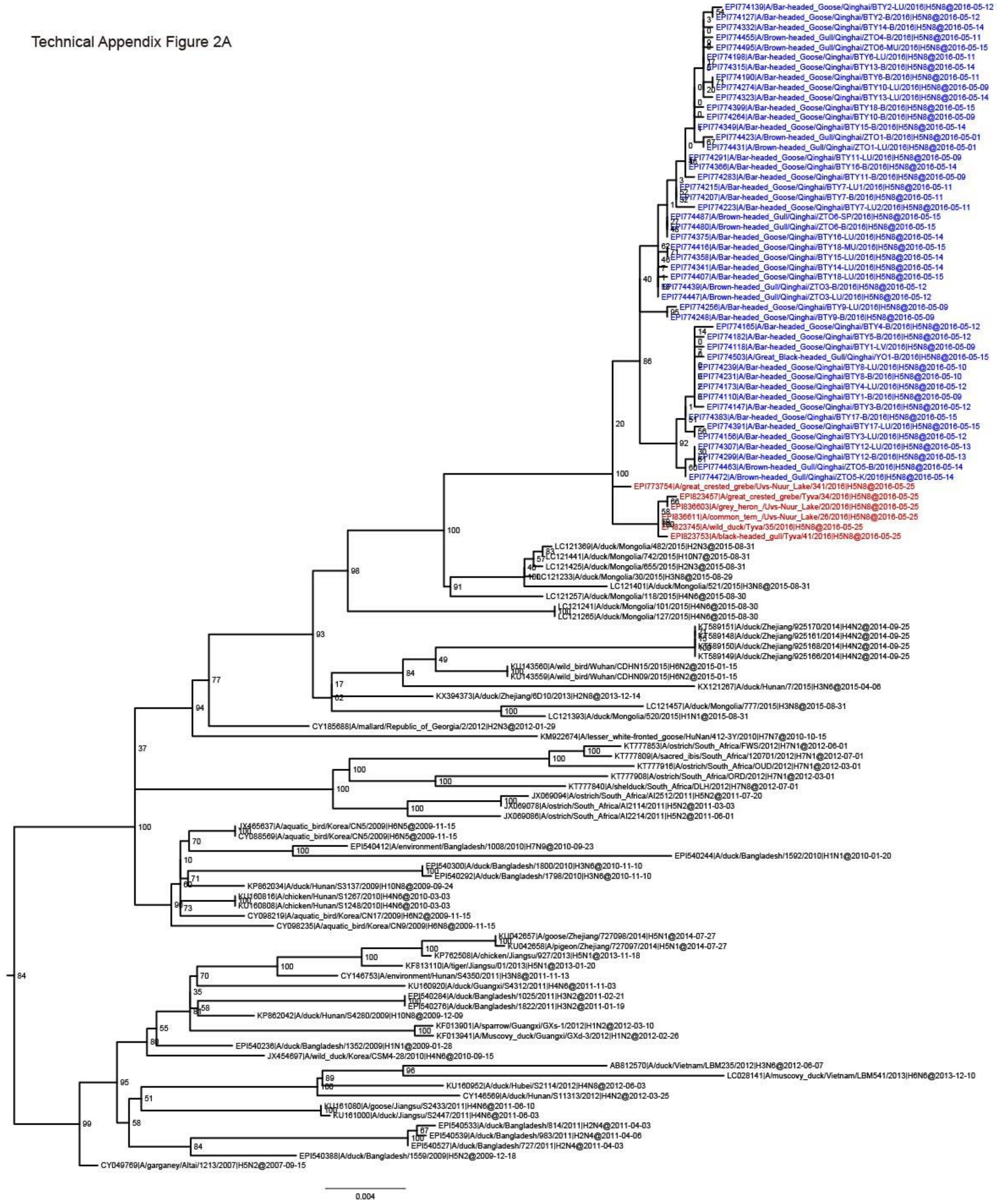

Technical Appendix Figure 2B

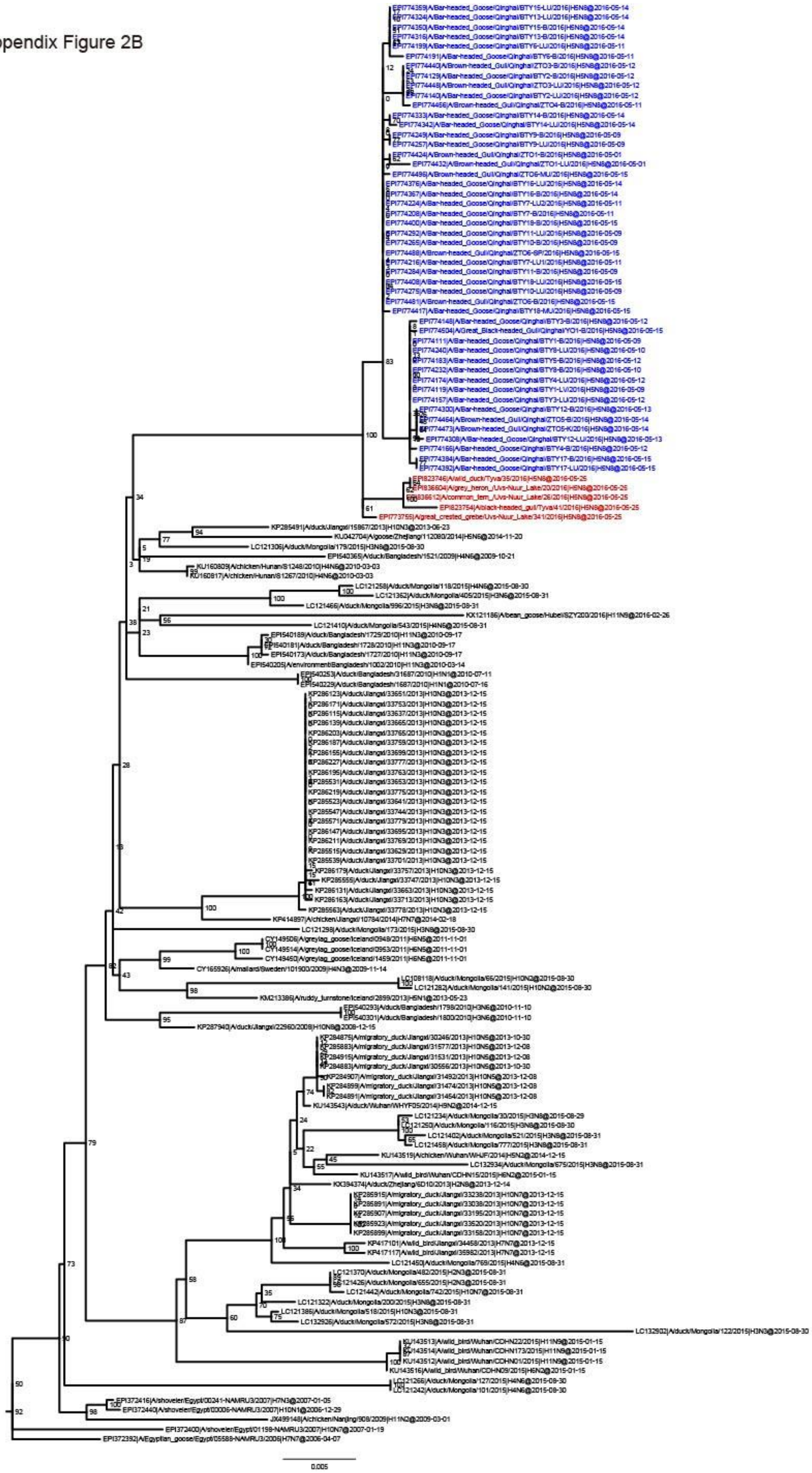

Technical Appendix Figure 2C

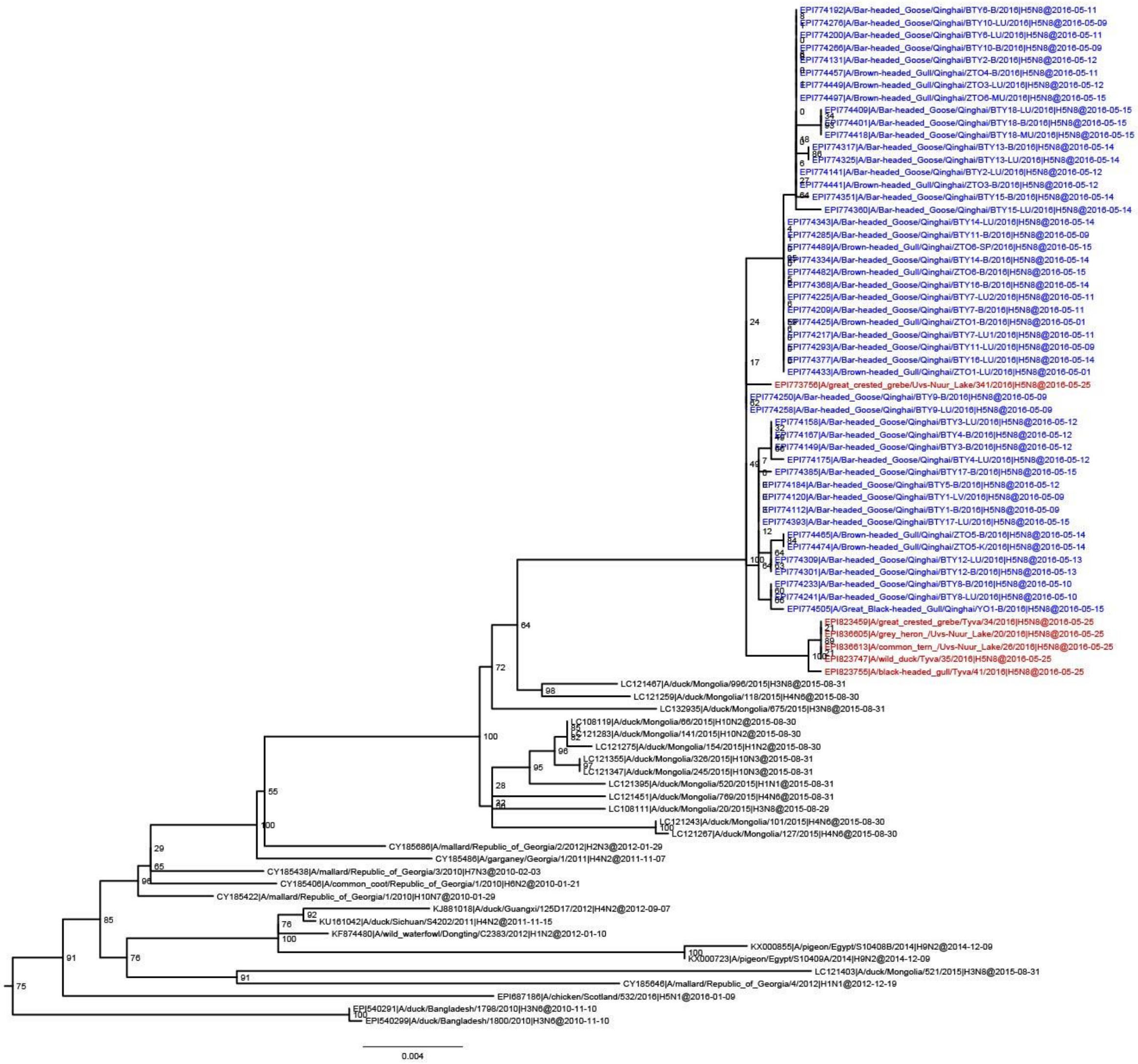

Technical Appendix Figure 2D

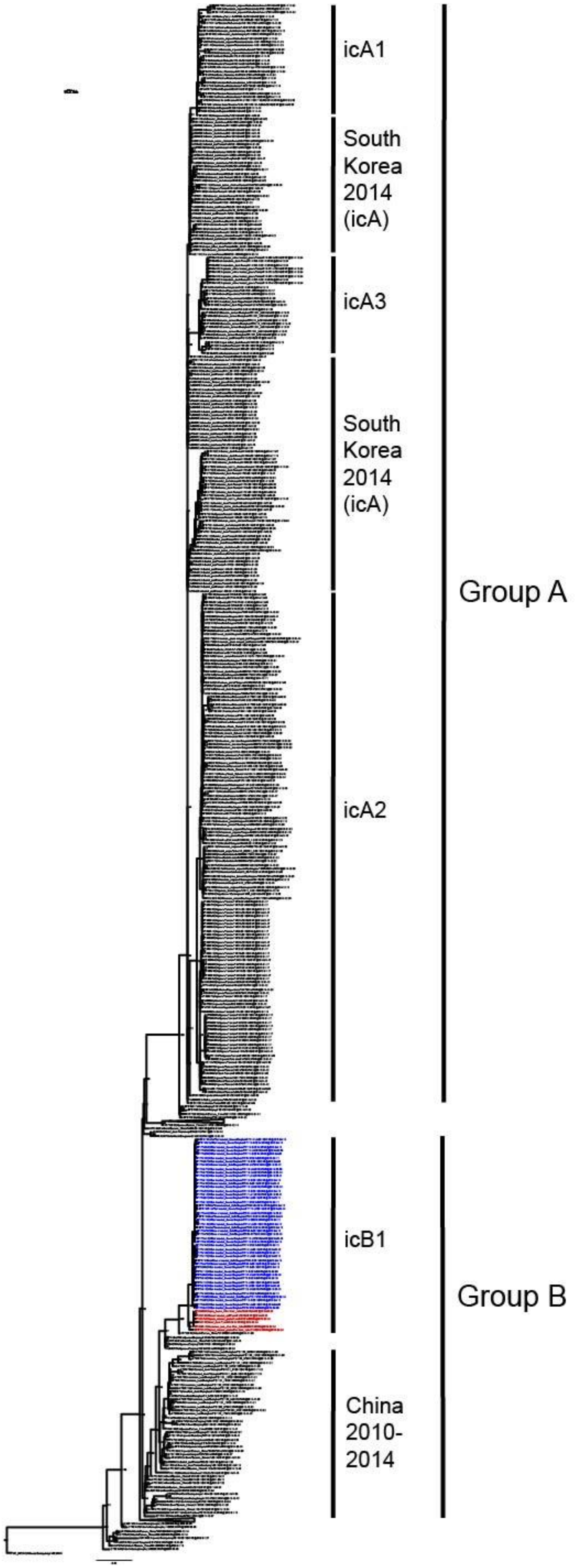

Technical Appendix Figure 2E

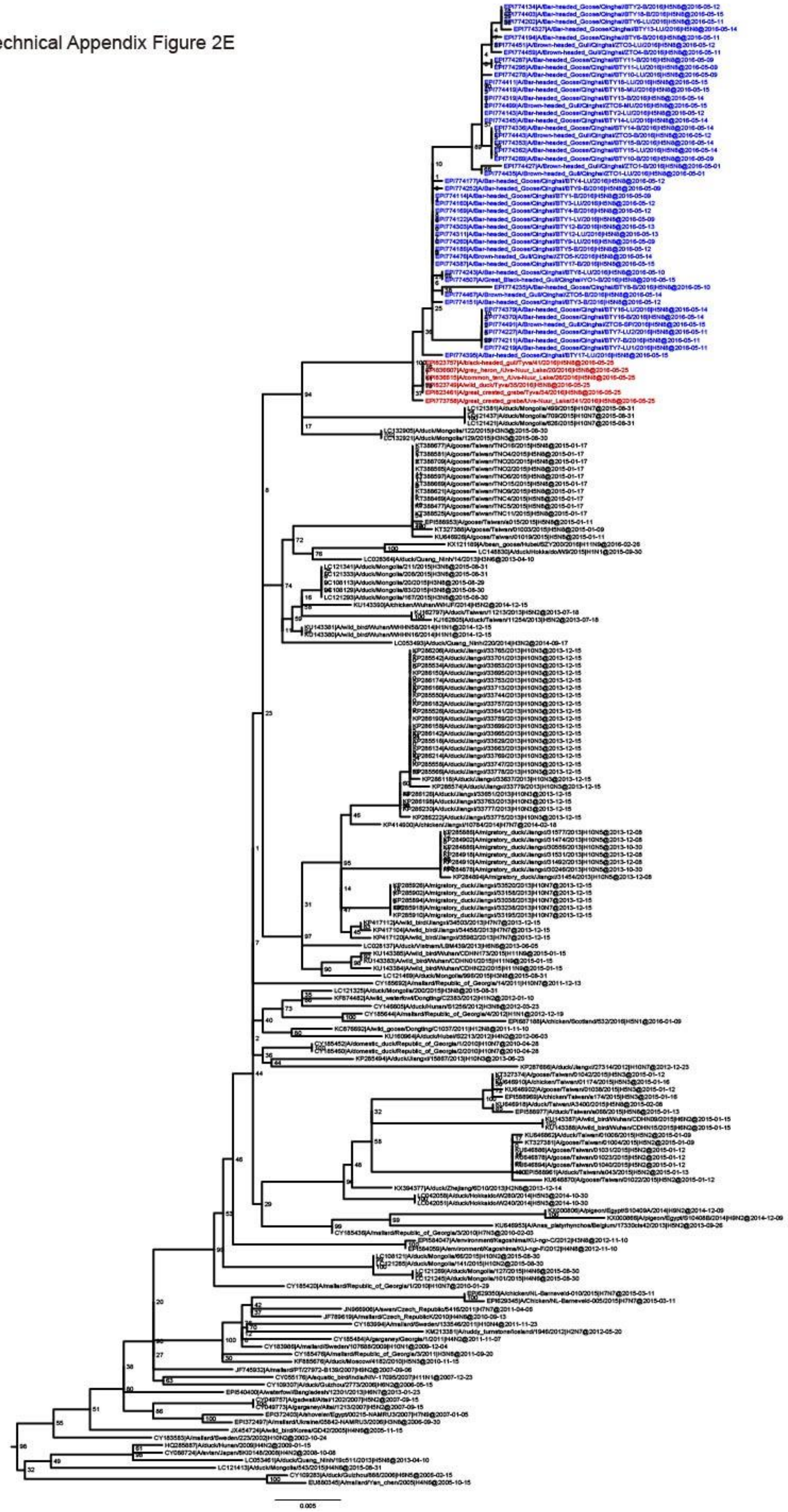

Technical Appendix Figure 2F

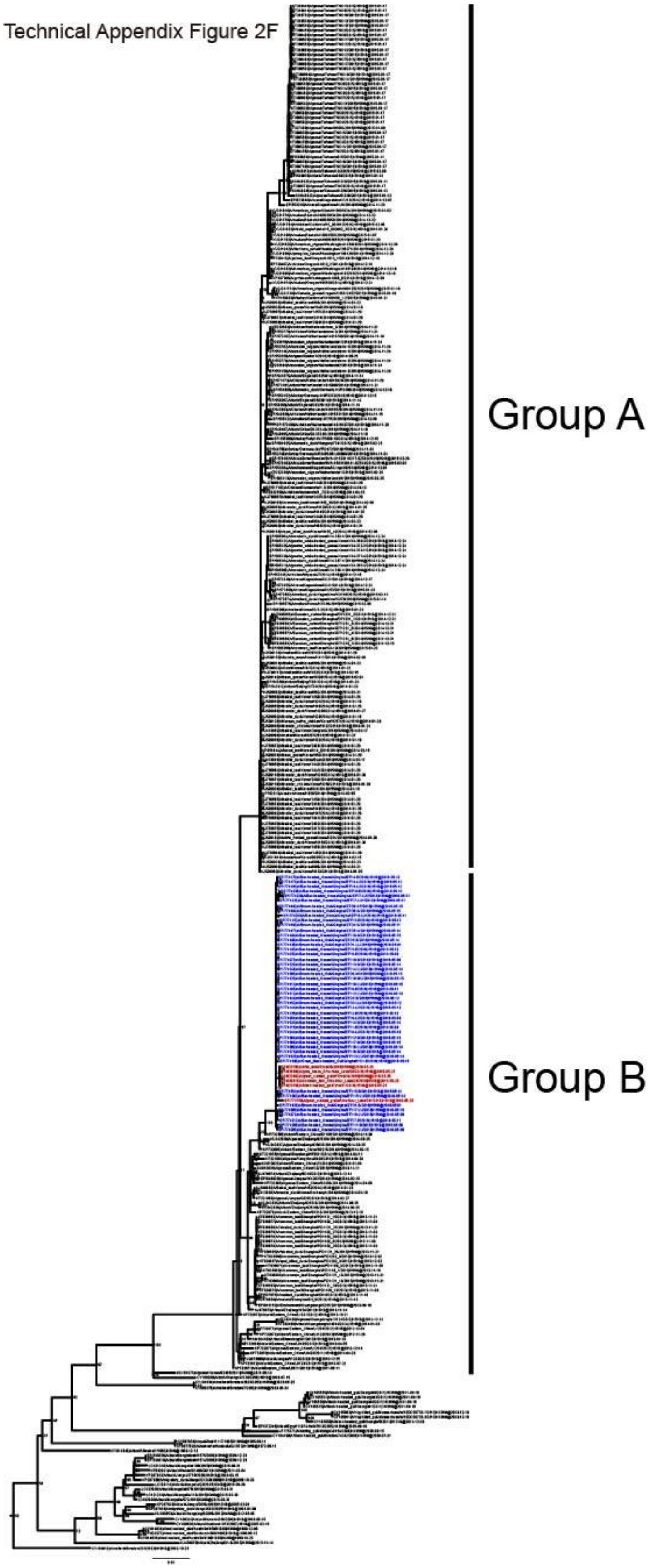

Technical Appendix Figure 2G

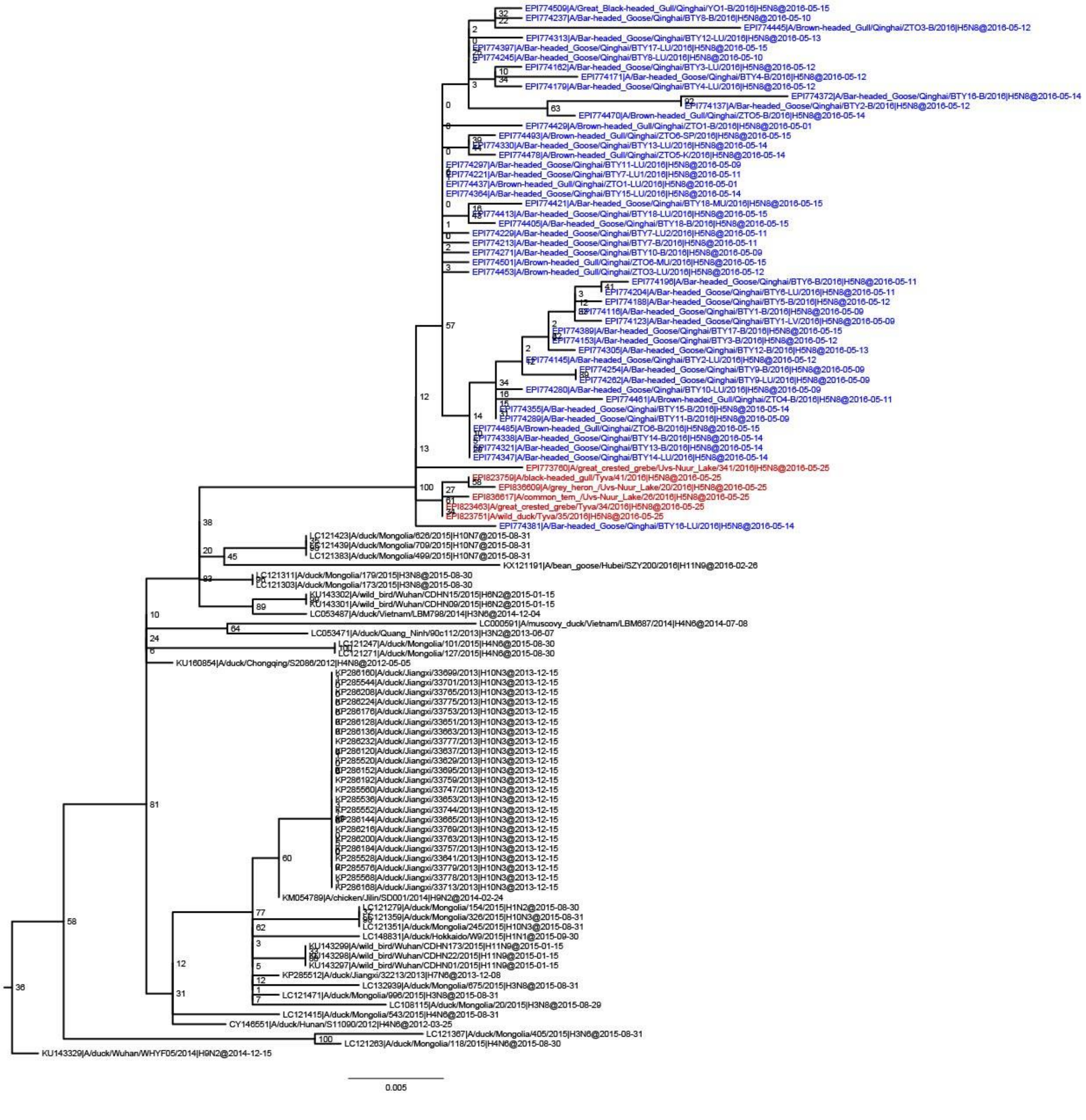

Technical Appendix Figure 2H

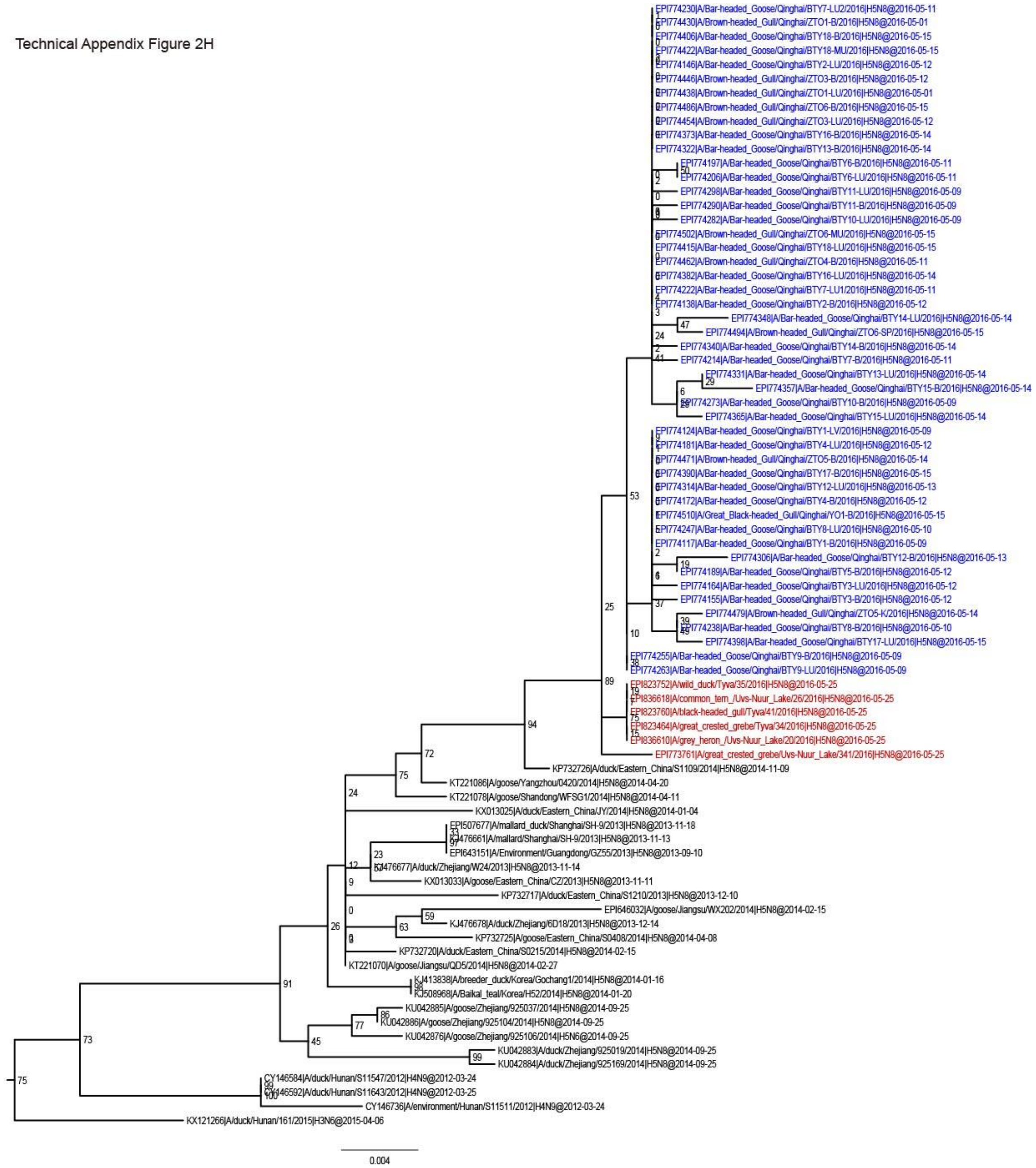

Technical Appendix Figure 2. Maximum-likelihood phylogenetic trees of the coding sequences of 8 segments. A) basic polymerase 2 (PB2), B) basic polymerase 1 (PB1), C) acidic polymerase (PA), D) hemagglutinin (HA), E) nucleoprotein (NP), F) neuraminidase (NA), G) matrix protein (MP), H) nonstructural protein (NS). Node labels indicate bootstrap values. Colored nodes are: blue, Qinghai Lake H5N8 strains (this study); red, Ubsu-Nur Lake H5N8 strains.

Technical Appendix Figure 3A

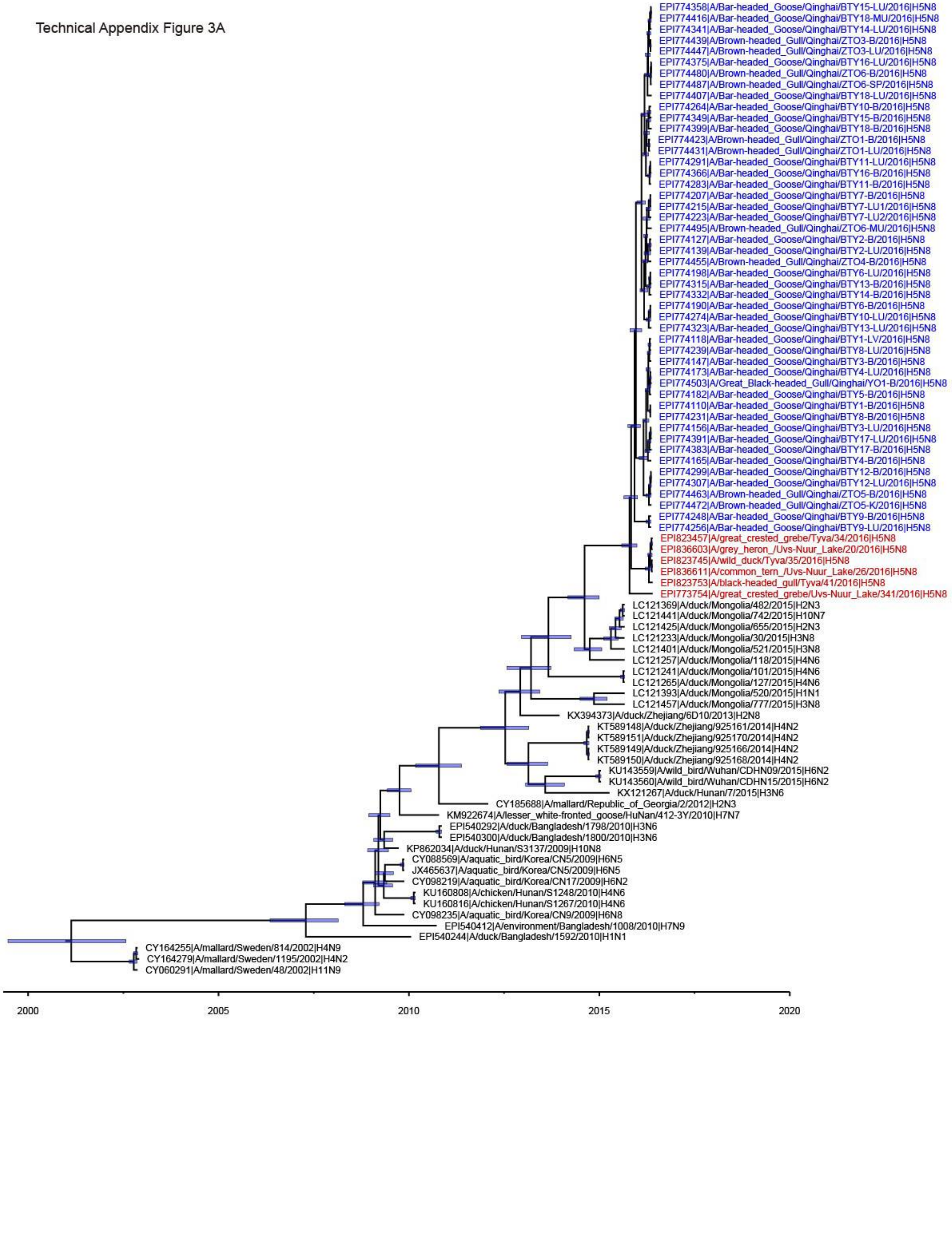

Technical Appendix Figure 3B

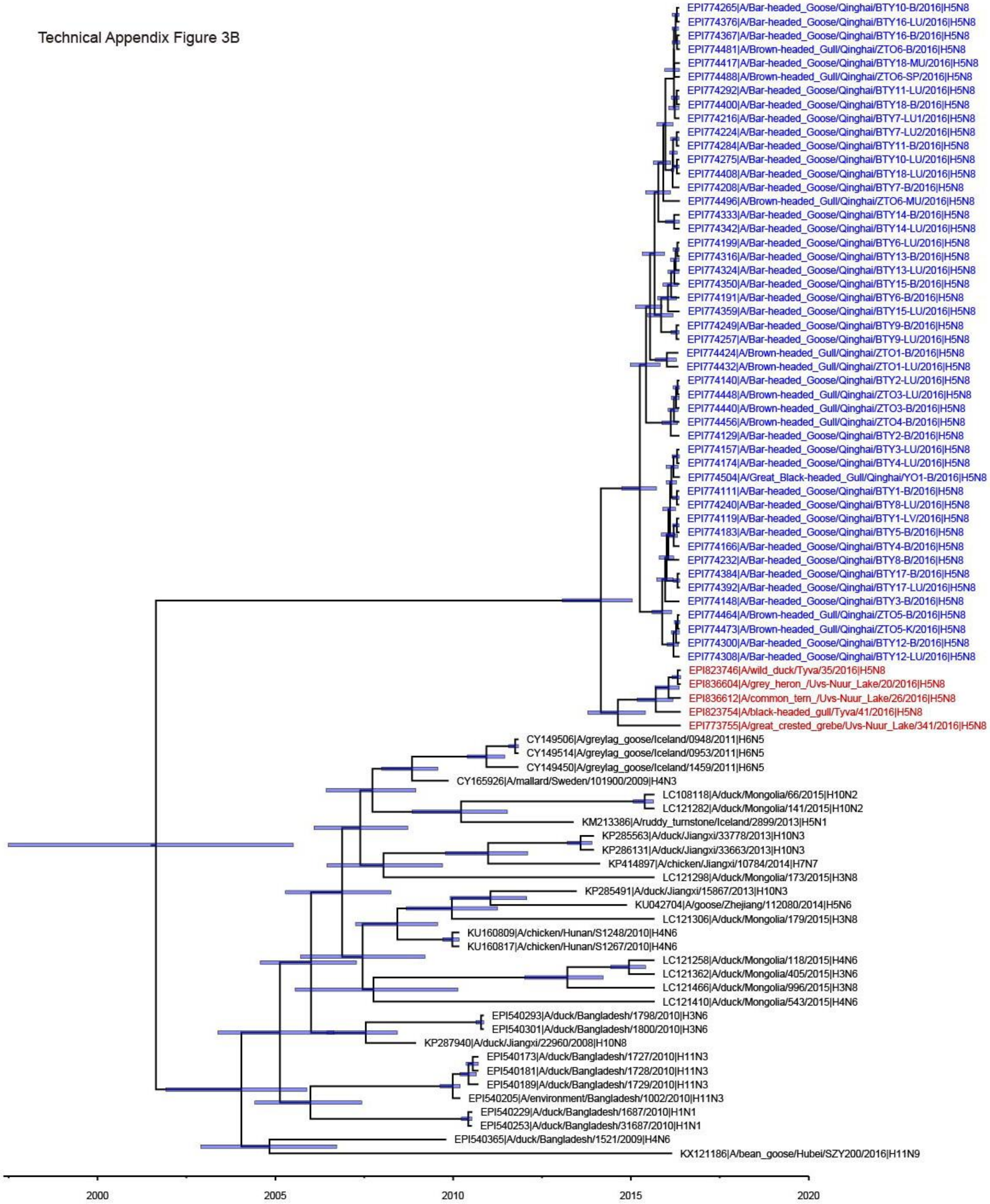

Technical Appendix Figure 3C

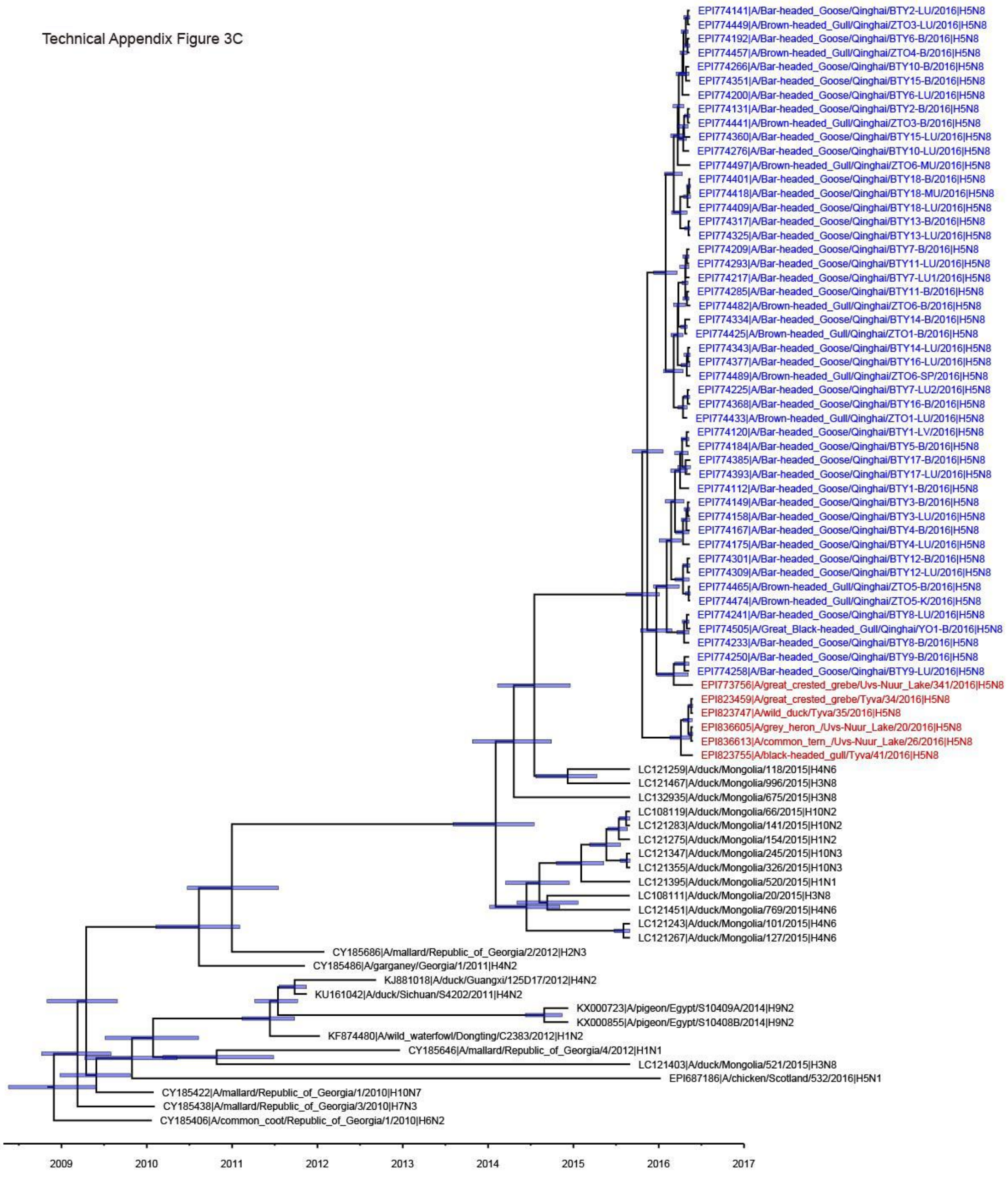

Technical Appendix Figure 3D

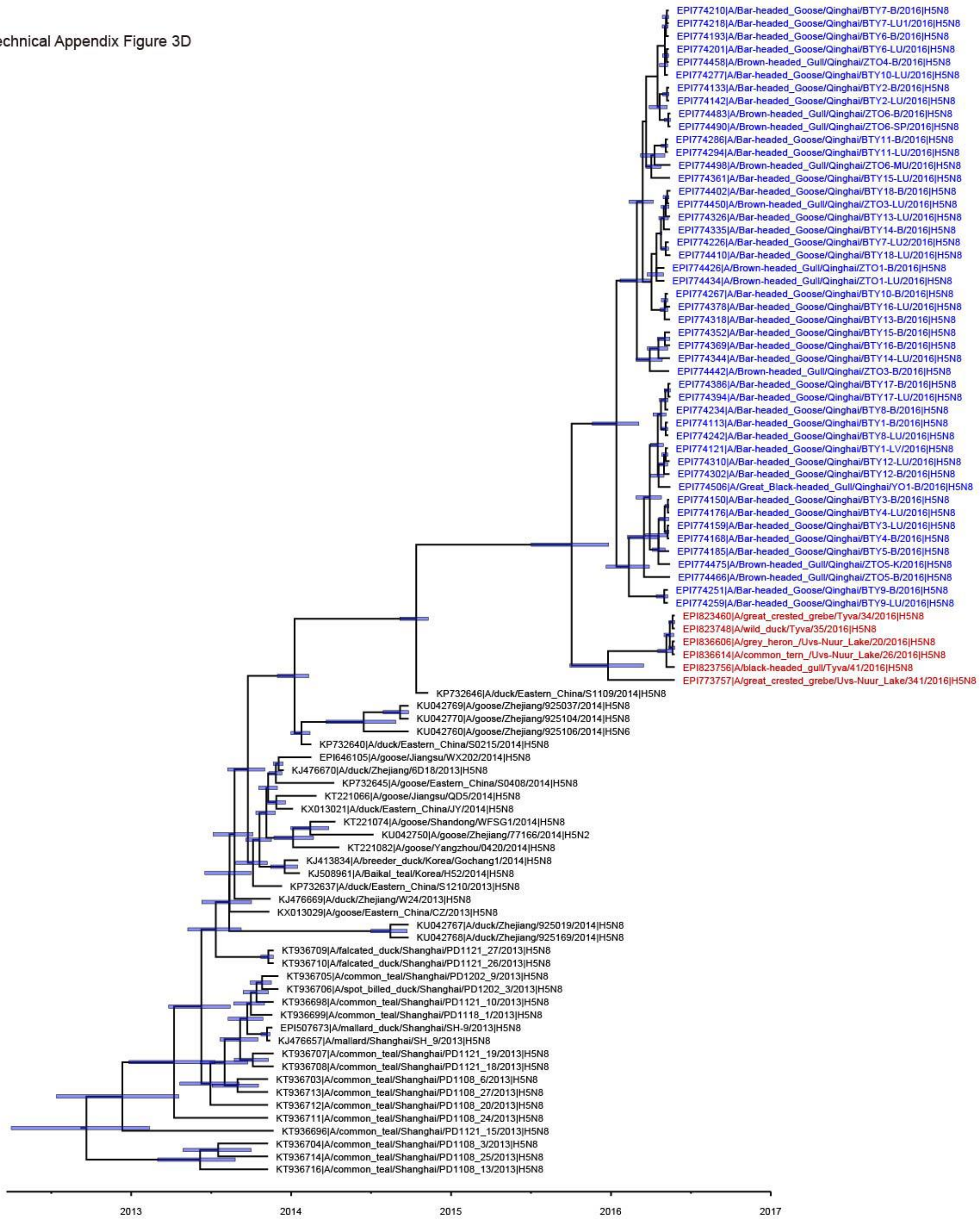

Technical Appendix Figure 3E

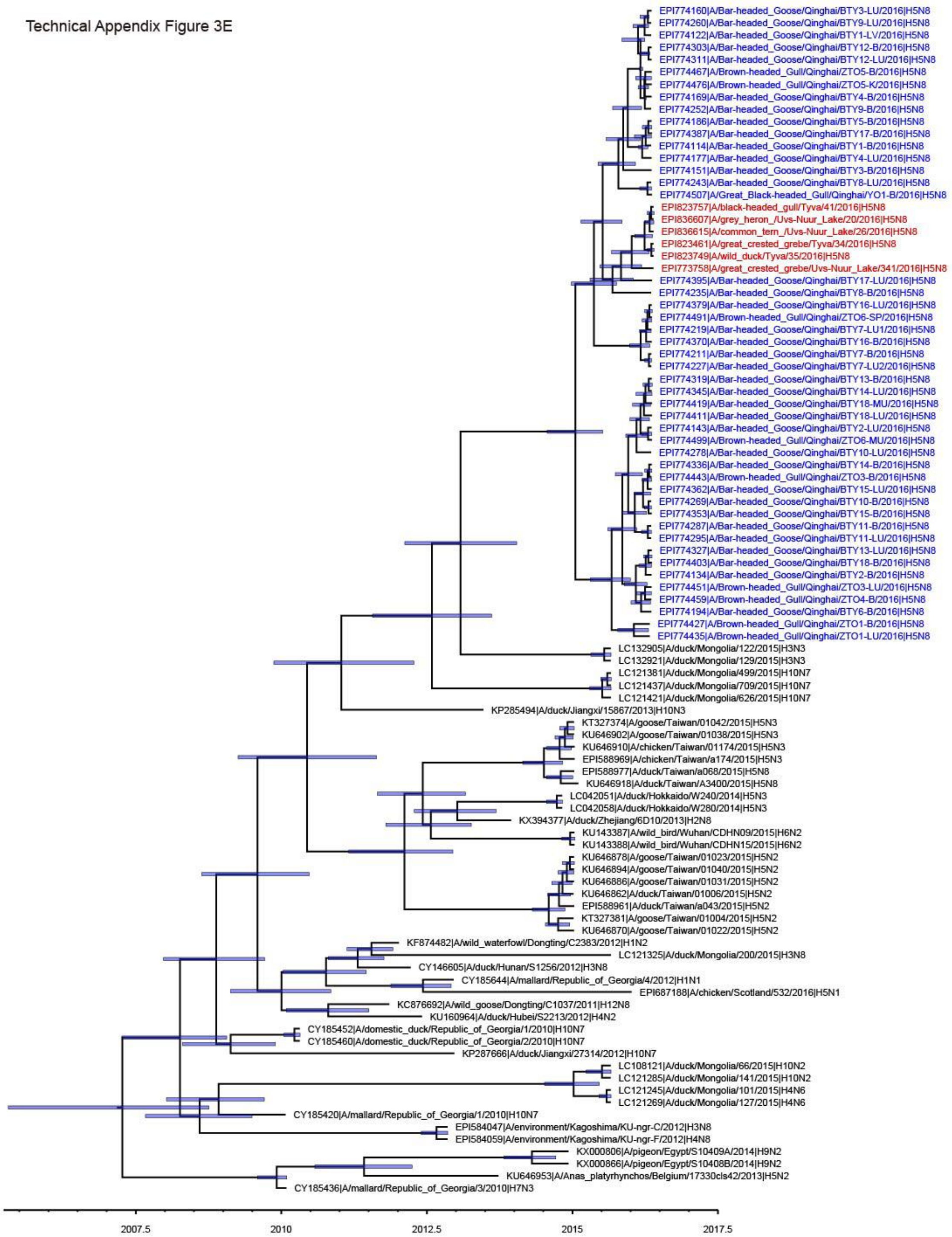

Technical Appendix Figure 3F

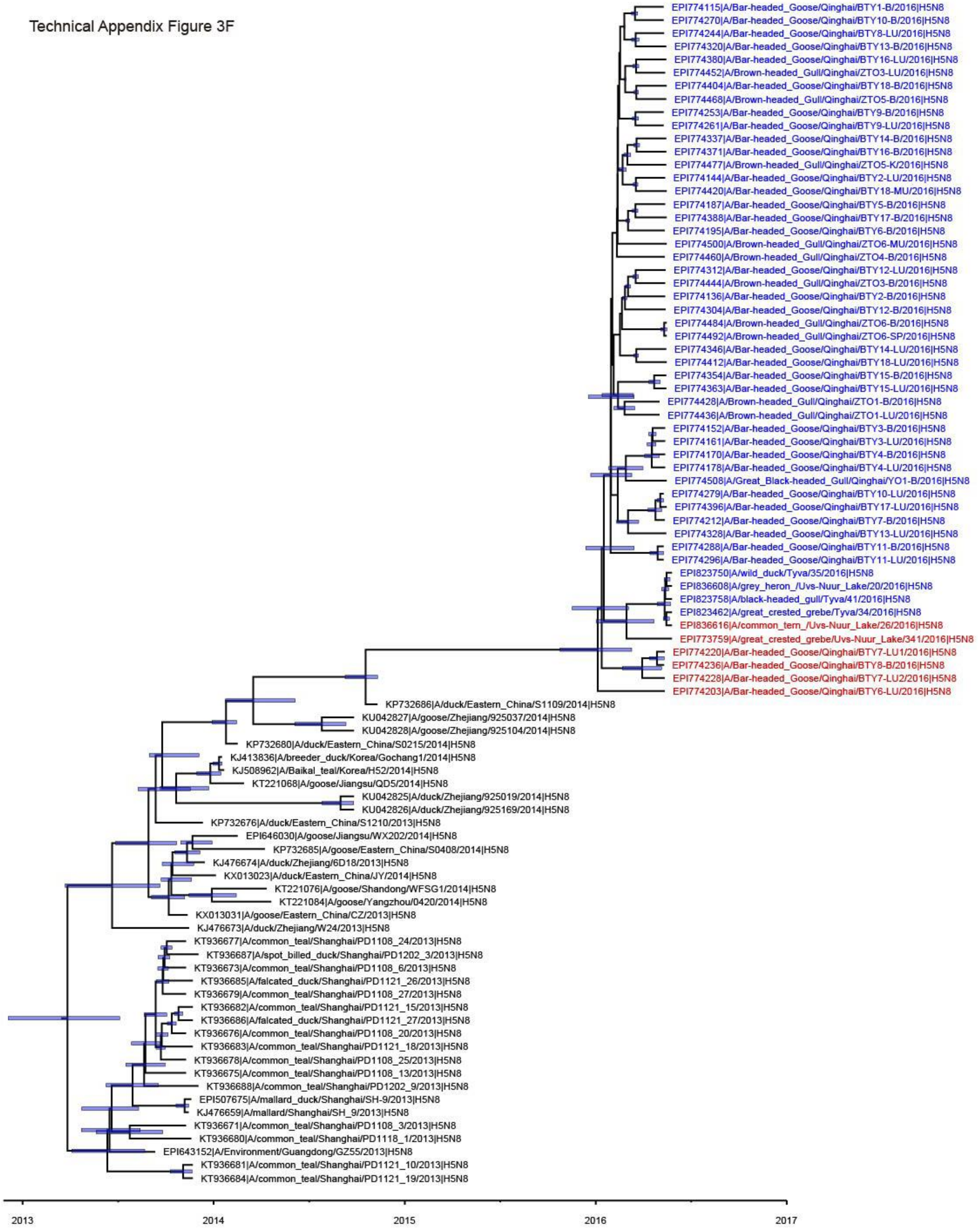

Technical Appendix Figure 3G

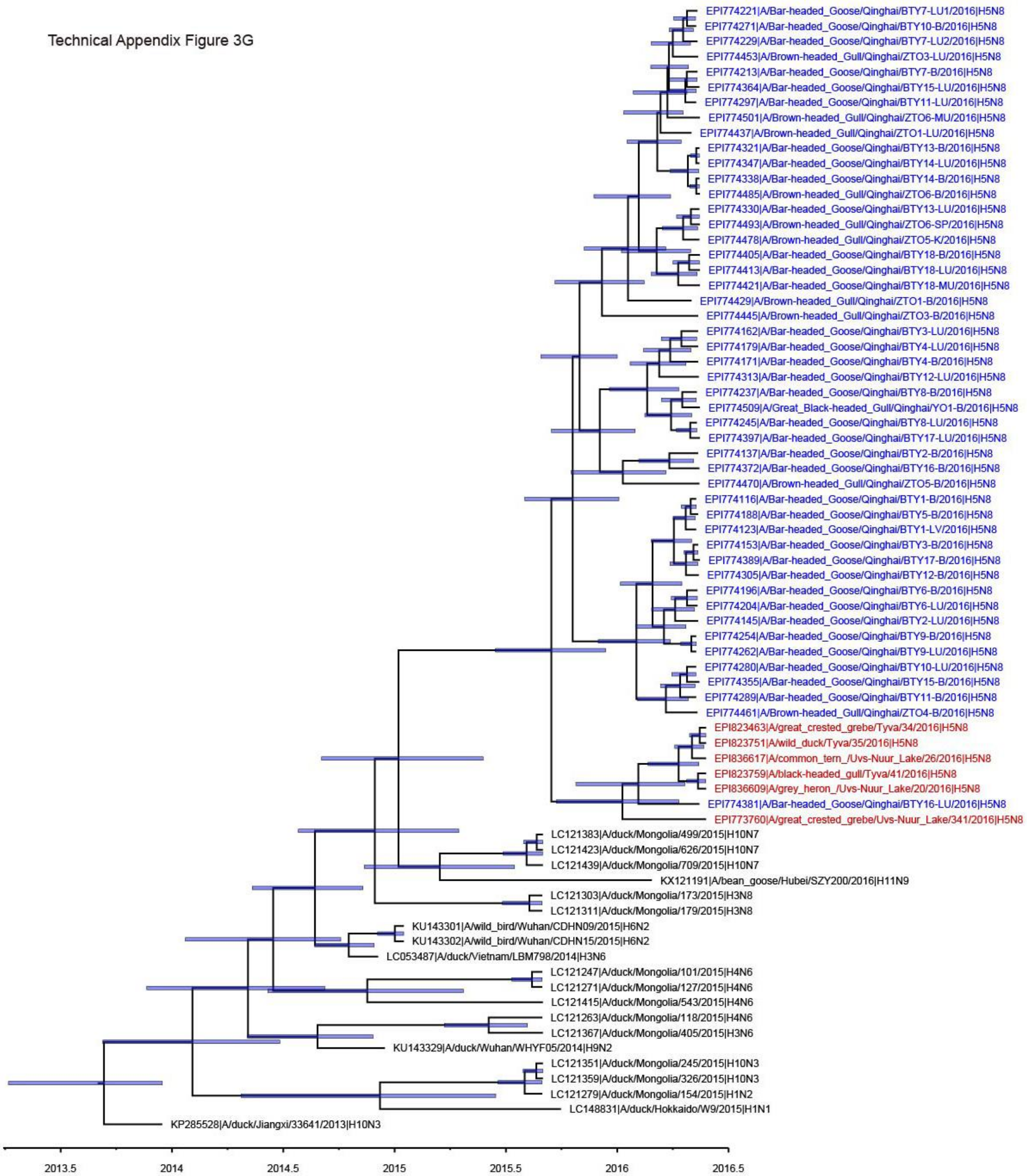

Technical Appendix Figure 3H

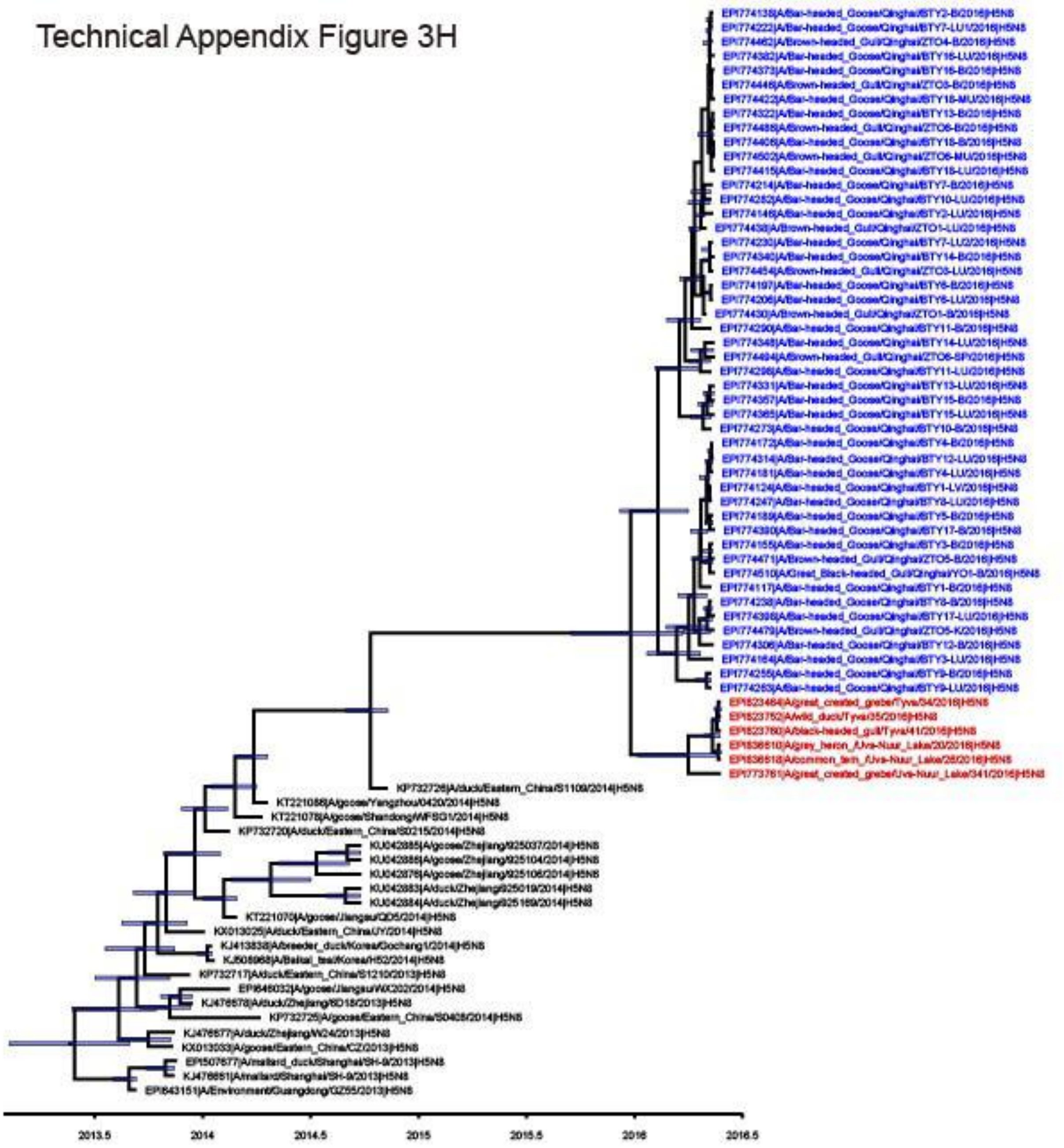

Technical Appendix Figure 3. Maximum clade credibility trees of the coding sequences of 8 segments: A) basic polymerase 2 (PB2), B) basic polymerase 1 (PB1), C) acidic polymerase (PA), D) hemagglutinin (HA), E) nucleoprotein (NP), F) neuraminidase (NA), G) matrix protein (MP), H) nonstructural protein (NS). Node bars indicate 95% highest posterior density (HPD) of the node height. Colored nodes are: blue, Qinghai Lake H5N8 strains (this study); red, Ubsu-Nur Lake H5N8 strains.
